# Assessment of virulence factors and antimicrobial resistance among the *Pseudomonas aeruginosa* strains isolated from animal meat and carcass samples

**DOI:** 10.1002/vms3.1007

**Published:** 2022-11-23

**Authors:** Shahrokh Poursina, Mohammad Ahmadi, Fatemeh Fazeli, Peiman Ariaii

**Affiliations:** ^1^ Department of Food Hygiene Ayatollah Amoli Branch Islamic Azad University Amol Iran; ^2^ Department of Food Science and Technology Ayatollah Amoli Branch Islamic Azad University Amol Iran

**Keywords:** *Pseudomonas aeruginosa*, prevalence, antibiotic resistance, virulence factors, raw meat, carcass surface swab

## Abstract

**Background:**

*Pseudomonas aeruginosa* bacteria are emerging causes of food spoilage and foodborne diseases. Raw meat of animal species may consider a reservoir of *P. aeruginosa* strains.

**Objectives:**

The present survey was done to assess the prevalence, antibiotic resistance properties and distribution of virulence factors among the *P. aeruginosa* strains isolated from raw meat and carcass surface swab samples of animal species.

**Methods:**

Five hundred and fifty raw meat and carcass surface swab samples were collected from cattle and sheep species referred to as slaughterhouses. *P. aeruginosa* bacteria were identified using culture and biochemical tests. The pattern of antibiotic resistance was determined by disk diffusion. The distribution of virulence and antibiotic resistance genes was determined using polymerase chain reaction.

**Results:**

Forty‐seven of 550 (8.54%) examined samples were contaminated with *P. aeruginosa*. The prevalence of *P. aeruginosa* in raw meat and carcass surface swab samples were 6.57 and 12%, respectively. *P. aeruginosa* isolates showed the maximum resistance rate toward penicillin (87.23%), ampicillin (85.10%), tetracycline (85.10%), gentamicin (65.95%) and trimethoprim (57.44%). The most commonly detected antibiotic resistance genes were *BlaCTX‐M* (53.19%), *blaDHA* (42.55%) and *blaTEM* (27.65%). The most commonly detected virulence factors was *ExoS* (42.55%), *algD* (31.91%), *lasA* (31.91%), *plcH* (31.91%) and *exoU* (25.53%).

**Conclusions:**

Meat and carcass surface swab samples may be sources of resistant and virulent *P. aeruginosa*, which pose a hygienic threat in their consumption. However, further investigations are required to identify additional epidemiological features of *P. aeruginosa* in meat and carcass surface samples.

## Graphical abstract

In the present study, the role of meat and animal carcasses as reservoirs of virulent and antibiotic‐resistant *Pseudomonas aeruginosa* strains was determined. *Pseudomonas aeruginosa* strains may transmit to the human population through the consumption of contaminated and uncooked meat.

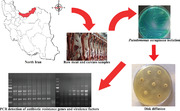

## 1 INTRODUCTION

Raw meat of animal species may act as a reservoir of several foodborne pathogens, responsible for both food spoilage and also the occurrence of foodborne diseases (Heredia & García, 2018). *Pseudomonas aeruginosa* (*P. aeruginosa*) is a food microorganism and opportunistic human pathogen extensively distributed in food and the environment (Gu et al., 2016). It is usually present in environmental sources, including soil and water. It is supposed that it can be found on vegetables, fruits and meat. Meat storage under aerobic conditions allows *P. aeruginosa* growth and proliferation even in different temperatures (Neto et al., 2012). It can easily develop in milk, fish, meat and dairy samples stored aerobically at low temperature. The growth of the bacterium is responsible for off‐flavours, pigmentation, slime and malodour production in meat and derived products (Stellato et al., 2017). Previous studies showed that drinking water contaminated with *P. aeruginosa* may cause foodborne infections (Wei et al., 2020; Vukić Lušić et al., 2021). Additionally, there is growing evidence implication of the *P. aeruginosa* in foodborne infections (Bricha et al., 2009; Nawaz & Bhattarai, 2015; Virupakshaiah & Hemalata, 2016). However, there is no available data about the occurrence of foodborne diseases after the consumption of meat contaminated with *P. aeruginosa*. Thus, there is a crucial need to determine the role of raw meat of animal species as a reservoir of *P. aeruginosa*.

*P. aeruginosa* contained several types of virulence factors responsible for the pathogenesis of diseases and related infections. Phenazine operons (*phzH*, *phzM* and *phzS*) secrete the precursor proteins to encode three phenazine compounds responsible for the intracellular oxidative effects (Heggins et al., 2018). Elastase gene A and B (*lasA* and *lasB*), exoenzymes (*exoS*, *exoT*, *exoU* and *exoY*), haemolytic and non‐haemolytic Phospholipase C (*plcH* and *plcN*) and alginate‐encoded genes (*algD* and *algU*) are other essential virulence factors of the *P. aeruginosa* (Rocha et al., 2019; Veetilvalappil et al., 2022). *P. aeruginosa* strains containing these genes can easily attach to epithelial cells and make invasion and further inflammation and injury (Moissenet & Khedher, 2011).

Recent reports revealed the high resistance rate of *P. aeruginosa* strains toward different types of antimicrobial agents (Pang et al., 2019; Soares et al., 2020). Antibiotic‐resistant *P. aeruginosa* strains caused more severe infections for a longer time with a higher economic burden (Meliani 2020). In 2017, resistant *P. aeruginosa* was responsible for about 32,500 infections and 2700 deaths in hospitalised patients in the United States (Kunz Coyne et al., 2022). In this regard, the highest resistance rates were reported against aminoglycosides, tetracyclines, penicillins, quinolones, cephalosporins, macrolides and β‐lactams antibiotics (Langendonk et al., 2021). Throughout the last years, extended‐spectrum b‐lactamases (ESBLs) types have arisen in the hospital and community setting among *P. aeruginosa* strains on plasmids that commonly stand as additional resistance determinants (Rahimi et al., 2021). *P. aeruginosa* resistance to cephalosporins, monobactam and carbapenems may be acquired by ESBL enzymes. Among them, *blaSHV*, *blaTEM*, *blaDHA*, *blaOXA*, *blaVEB* and *blaCTX‐M* genes are the most widespread and clinically relevant (APHA 2001; Abdelrahman et al., 2020).

According to the uncertain role of meat and animal carcasses as sources of *P. aeruginosa* transmission to the human population and also a risk of foodborne diseases, the present study was done to assess the prevalence and antibiotic resistance rate and distribution of virulence factors and antibiotic resistance genes of *P. aeruginosa* strain isolated from raw meat and carcasses surface swab samples of bovine and ovine species.

## 2 MATERIALS AND METHODS

### 2.1 Study area

**FIGURE 1:**
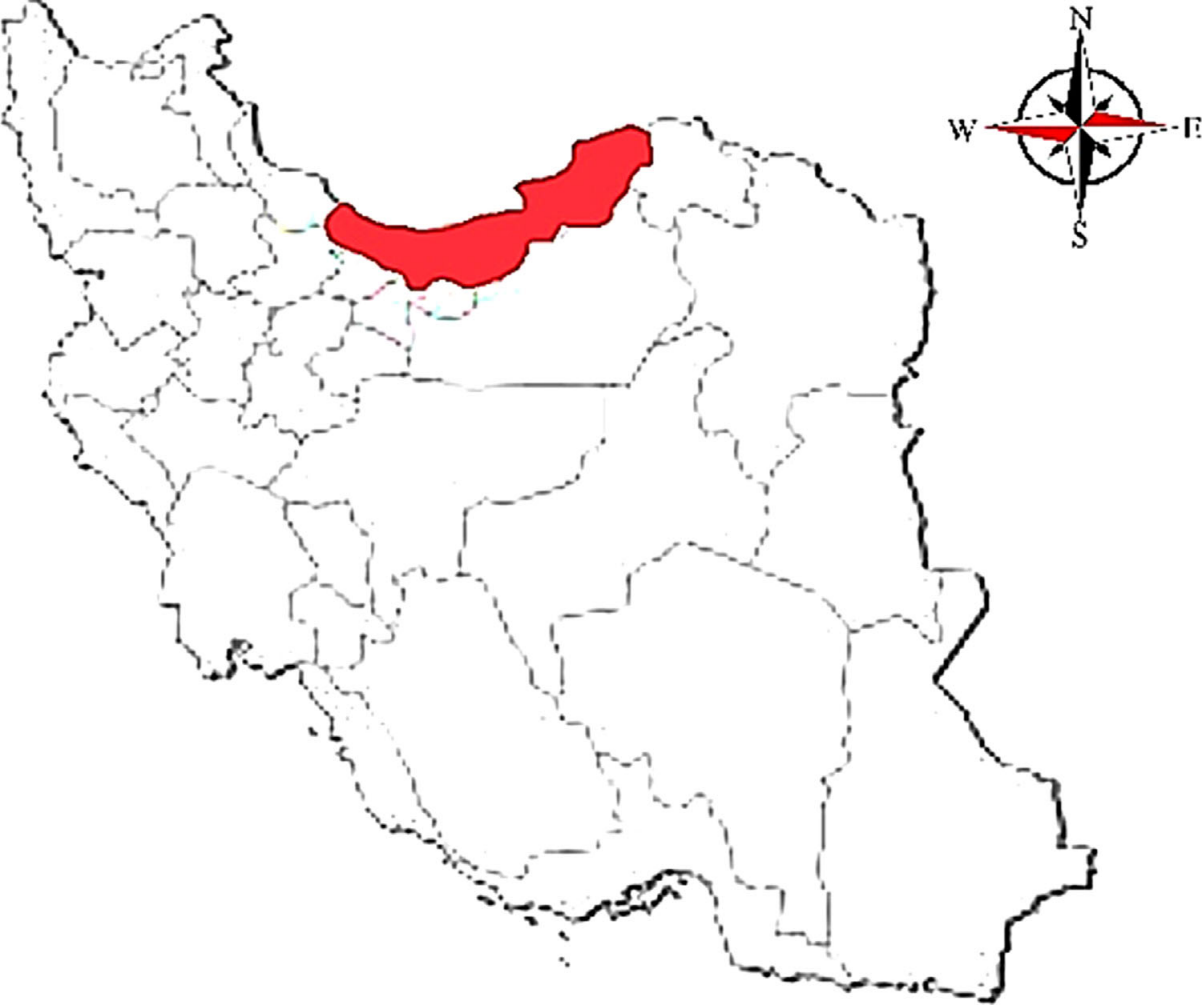
Map of the study area

Figure 1 shows the map of the study area. The study was conducted from November 2021 to March 2022 in Mazandaran and Golestan province, North of Iran. Golestan province is located at 55.1376°E and 37.2898°N at an average altitude of 3820 m. a. s. l. and 510 km far from Tehran. The climate of this province is mainly humid and rainy. The mean annual rainfall and temperature of Golestan province are 609 mm and 17.6°C, respectively. Mazandaran province is located at 36.2262°N, 52.5319°E at an average altitude of 1500–3000 m. a. s. l. (depends on location) and 189.2 km far from Tehran. The climate of this province is mainly humid and rainy. The mean annual rainfall and temperature of Mazandaran province are 977 mm and 18.3°C, respectively. Different traditional slaughterhouses exist in this region. Slaughterhouses in this area are usually traditional and animal slaughtering is sometimes done without following hygienic protocols.

### 2.2 Samples

The sample size was determined using the following formula. A total of 550 samples, including raw cattle (*n* = 175) and sheep (*n* = 175) meat and cattle (*n* = 100) and sheep (*n* = 100) carcasses surface swab samples were randomly collected from animals referred to slaughterhouses in North Iran. Raw meat samples (100 g) were collected from the tight muscle using sterile plastic bags. Carcass swab samples were taken from a 20 cm^2^ area of the tight muscle after bleeding, skinning, eviscerating and washing stages using the swabbing technique. Swab samples were transferred using sterile tubes containing 0.1% peptone water solution. Samples were transferred in refrigerated containers at 4°C. Samples transportation and processing were done within 2 h after collection.

$$\;N=Z^{2}\frac{pq}{d^{2}}$$

where *p* is the *H. pylori* mean prevalence in recent studies, *Z* is the abscissa of the standard curve that cuts off an area *α* at the tails (1.96), *d* is the acceptable sampling error and *N* is the sample size.

### 2.3 *P. aeruginosa* isolation and identification

Twenty‐five grams of the collected meat samples were put in sterile Stomacher bags containing 225 ml peptone water (Oxoid, Basingstoke, UK). The bags underwent maceration within the stomacher (Seward 400 circulator) for 4 min at 260 beats per min. A total of 100 µl homogenate meat and also swab solution samples were placed on CN Selective Agar (Oxoid SR 102E) supplemented with Pseudomonas Agar base (Oxoid). Media were incubated at 37°C for 24 h in aerobic conditions. Pseudomonas were identified by microscopic morphology, catalase, oxidase and urease activity, casein and starch hydrolysis, citrate and indole utilisation and Methyl Red–Voges Proskauer and gelatin liquefaction tests, using standard microbial techniques. Additionally, API 20NE strips (BioMeriouxVitek, Inc., MO, USA) system was used to identify *P. aeruginosa*. *P. aeruginosa* isolates were identified another time using the polymerase chain reaction (PCR)‐based detection of *16S rRNA* specific gene (F: 5′‐GGGGGATCTTCGGACCTCA‐3′ and R: 5′‐TCCTTAGAGTGCCCACCCG‐3′ (956 bp)) (Spilker et al., 2004).

### 2.4 Antimicrobial resistance analysis

To investigate the pattern of antibiotic resistance of *P. aeruginosa* isolates, the simple disk diffusion method (Kirby Baeur) was used. *P. aeruginosa* isolates were cultured overnight in Trypton Soya broth (TSB, Merck, Germany) medium and incubated in an aerobic condition at 37°C for 24 h. After the preparation of 0.5 McFarland concentration (a bacterial suspension containing between 1 × 10^8^ and 2 × 10^8^ CFU/ml of bacteria), isolates were cultured on the Mueller–Hinton agar (Merck, Germany) in the presence of diverse antibiotic discs, including tetracycline (30 µg/disk), chloramphenicol (30 µg/disk), sulfamethoxazole (25 µg/disk), gentamicin (10 µg/disk), ciprofloxacin (5 µg/disk), trimethoprim (5 µg/disk), ampicillin (10 µg/disk), penicillin (10 µg/disk), cefoxitin (10 µg/disk), clindamycin (2 µg/disk), imipenem (10 µg/disk) and aztreonam (30 µg/disk) (Oxoid) were placed on media. All media were incubated at 37°C for 24 h. The antibiotic resistance pattern of isolates was assessed by measuring the diameter of the growth inhibition halo around each disk, and the susceptibility or resistance to the relevant antibiotic was determined and recorded. In this experiment, *P. aeruginosa* standard strain (ATCC 10145) was used as a positive control. Findings were interpreted rendering the Antimicrobial Susceptibility Testing European Committee (EUCAST, 2018) CLSI criteria (CLSI, 2016, CLSI, 2018, Dehkordi et al., 2017).

### 2.5 DNA extraction and quality analysis

Identified *P. aeruginosa* isolates were sub‐cultured on TSB media (Merck, Germany) and incubated for 48 h at 37°C in an aerobic condition. According to the manufacturer's instructions, the genomic DNA was extracted from the isolates using the DNA extraction kit (Thermo Fisher Scientific, St. Leon‐Rot, Germany). The purity (A260/A280) and concentration of the extracted DNA were then checked (NanoDrop; Thermo Scientific, Waltham, MA, USA). Furthermore, the DNA's quality was assessed on a 2% agarose gel stained with ethidium bromide (0.5 µg/ml) (Thermo Fisher Scientific, St. Leon‐Rot, Germany) (Dehkordi et al., 2020).

### 2.6 Assessment of antibiotic resistance genes and virulence factors

Table 1 shows the PCR conditions met to detect antibiotic resistance genes and virulence factors (Oliver et al., 2002; Pagani et al., 2003; Finnan et al., 2004; Pai et al., 2004; Gorgani et al., 2009). A programmable DNA thermocycler (Eppendorf Mastercycler 5330; Eppendorf‐Nethel‐Hinz GmbH, Hamburg, Germany) was used in all PCR reactions. In addition, amplified samples were analysed by electrophoresis (120 V/208 mA) in a 2.5% agarose gel stained with 0.1% ethidium bromide (0.4 µg/ml) (Rahi et al., 2020). Besides, UVI doc gel documentation systems (Grade GB004; Jencons PLC, London, UK) were used to analyse images (Ranjbar et al., 2019).

**TABLE 1:** PCR conditions used to detect virulence factors and antibiotic resistance genes among *P. aeruginosa* isolates (Oliver et al., 2002; Pagani et al., 2003; Finnan et al., 2004; Pai et al., 2004; Gorgani et al., 2009)

Targeted genes	Primer sequence (5′–3′)	PCR product (bp)	PCR programs	PCR volume (50 µl)
*algD*	F:AAGGCGGAAATGCCATCTCC R: AGGGAAGTTCCGGGCGTTTG	275	1 cycle: 2 min: 95°C 30 cycles: 30 s: 94°C 30 s: 58°C 60 s: 72°C 1 cycle: 7 min: 72°C	10× PCR buffer: 5 µl Mgcl_2:_ 1.5 mM dNTP: 200 µM Primer F: 0.5 µM Primer R: 0.5 µM Taq DNA polymerase: 1.25 U DNA: 2.5 µl
*algU*	F: CGCGAACCGCACCATCGCTC R: GCCGCACGTCACGAGC	410		
*lasB*	F: ACACAATACATATCAACTTCGC R: AGTGTGTTTAGAATGGTGATC	284	1 cycle: 3 min: 94°C 30 cycles: 30 s: 94°C 60 s: 55°C 90 s: 72°C 1 cycle: 5 min: 72°C	10× PCR buffer: 5 µl Mgcl_2:_ 1.5 mM dNTP: 200 µM Primer F: 0.5 µM Primer R: 0.5 µM Taq DNA polymerase: 1.25 U DNA: 2.5 µl
*plcH*	F: CACACGGAAGGTTAATTCTGA R: CGGTTARACGGCTGAACCTG	608		
*plcN*	F: CGACTTCCATTTCCCGATGC R: GGACTCTGCAACAAATACGC	481		
*exoS*	F: GTGTGCTTTATGCCATGAG R: GGTTTCCTTTTCCAGGTC	444		
exoT*	F: CAATCATCTCAGCAGAACCC R: TGTCGTAGAGGATCTCCTG	1159	1 cycle: 5 min: 96°C 30 cycles: 30 s: 94°C 30 s: 47–63°C 1 min: 72°C 1 cycle: 3 min: 72°C	10× PCR buffer: 5 µl Mgcl_2:_ 1.5 mM dNTP: 200 µM Primer F: 0.5 µM Primer R: 0.5 µM Taq DNA polymerase: 1.25 U DNA: 2.5 µl
*exoY*	F: TATCGACGGTCATCGTCAGGT R: TTGATGCACTCGACCAGCAAG	1035		
*exoU*	F: GATTCCATCACAGGCTCG R: CTAGCAATGGCACTAATCG	3308		
*phzM*	F: ATGGAGAGCGGGATCGACAG R: ATGCGGGTTTCCATCGGCAG	875		
*phzS*	F: TCGCCATGACCGATACGCTC R: ACAACCTGAGCCAGCCTTCC	1752		
*phzH*	F: GCCAAGGTTTGTTGTCGG R: CGCATTGACGATATGGAAC	1036		
*lasA*	F: GCAGCACAAAAGATCCC R: GAAATGCAGGTGCGGTC	1075		
*blaTEM*	F: ATGAGTATTCAACATTTCCG R: CTGACAGTTACCAATGCTTA	867	1 cycle: 5 min: 96°C 35 cycles: 60 s: 96°C 60 s: 58°C 60 s: 72°C 1 cycle: 10 min: 72°C	10× PCR buffer: 5 µl Mgcl_2:_ 1.5 mM dNTP: 200 µM Primer F: 0.5 µM Primer R: 0.5 µM Taq DNA polymerase: 1.25 U DNA: 2.5 µl
*blaSHV*	F: GGTTATGCGTTATATTCGCC R: TTAGCGTTGCCAGTGCTC	867	1 cycle: 5 min: 96°C 35 cycles: 60 s: 96°C 60 s: 60°C 60 s: 72°C 1 cycle: 10 min: 72°C	10× PCR buffer: 5 µl Mgcl_2:_ 1.5 mM dNTP: 200 µM Primer F: 0.5 µM Primer R: 0.5 µM Taq DNA polymerase: 1.25 U DNA: 2.5 µl
*blaOXA*	F: ACACAATACATATCAACTTCGC R: AGTGTGTTTAGAATGGTGATC	885		
*blaCTX‐M*	F: ATGTGCAGYACCAGTAARGT R: TGGGTRAARTARGTSACCAGA	593	1 cycle: 7 min: 94°C 35 cycles: 50 s: 94°C 40 s: 50°C 60 s: 72°C 1 cycle: 5 min: 72°C	10× PCR buffer: 5 µl Mgcl_2:_ 1.5 mM dNTP: 200 µM Primer F: 0.5 µM Primer R: 0.5 µM Taq DNA polymerase: 1.25 U DNA: 2.5 µl
*blaDHA*	F: CACACGGAAGGTTAATTCTGA R: CGGTTARACGGCTGAACCTG	970	1 cycle: 5 min: 94°C 35 cycles: 30 s: 94°C 45 s: 50°C 60 s: 72°C 1 cycle: 8 min: 72°C	10× PCR buffer: 5 µl Mgcl_2:_ 1.5 mM dNTP: 200 µM Primer F: 0.5 µM Primer R: 0.5 µM Taq DNA polymerase: 1.25 U DNA: 2.5 µl
*blaVEB*	F: CGACTTCCATTTCCCGATGC R: GGACTCTGCAACAAATACGC	1014	1 cycle: 5 min: 96°C 30 cycles: 60 s: 96°C 60 s: 55°C 2 min: 72°C 1 cycle: 10 min: 72°C	10× PCR buffer: 5 µl Mgcl_2:_ 1.5 mM dNTP: 200 µM Primer F: 0.5 µM Primer R: 0.5 µM Taq DNA polymerase: 1.25 U DNA: 2.5 µl

*Annealing temperature of *exoT*, *exoY*, *exoU*, *phzM*, *phzS*, *phzH*, *lasA* and *lasB* virulence factors were 54, 61.8, 61.6, 54, 63, 51, 47 and 57°C, respectively.

### 2.7 Data analysis

Data analysis was performed by SPSS Statistics 21.0 (SPSS Inc., Chicago, IL, USA). Chi‐square and Fisher's exact two‐tailed tests were performed to assess any significant relationship between the *P. aeruginosa* prevalence and virulence and antibiotic resistance properties. Besides, *p* value < 0.05 was considered statistically significant (Dehkordi et al., 2017; Ranjbar et al., 2019).

## 3 RESULTS

### 3.1 *P. aeruginosa* prevalence

**FIGURE 2:**
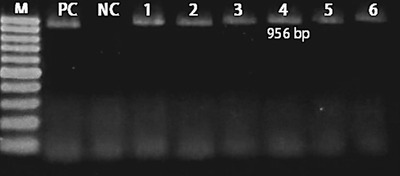
Gel electrophoresis of *16SrRNA* gene amplification in this PCR reaction. M, ladder (100 bp, Thermo Fisher Scientific, St. Leon‐Rot, Germany); PC, positive control; NC, negative control; 1–6, positive samples for *P. aeruginosa 16SrRNA* gene (956 bp)

Figure 2 shows the gel electrophoresis of 16SrRNA gene amplification in this PCR reaction.

Table 2 shows the *P. aeruginosa* prevalence among the examined meat and surface swab samples. Forty‐seven of 550 (8.54%) collected samples were contaminated with *P. aeruginosa*. *P. aeruginosa* prevalence among the raw meat and carcass surface swab samples were 6.57 and 12%, respectively. Samples collected from cattle had a higher contamination rate (*p* < 0.05). Statistically significant differences were observed between types of samples and *P. aeruginosa* contamination rate (*p* < 0.05).

**TABLE 2:** *P. aeruginosa* prevalence among the examined meat and surface swab samples

Types of samples	*N* collected samples	*N* (%) positive for *P. aeruginosa*
Raw cattle meat	175	13 (7.42)
Raw sheep meat	175	10 (5.71)
Total	350	23 (6.57)
Cattle carcass surface swab	100	14 (14)
Sheep carcass surface swab	100	10 (10)
Total	200	24 (12)
Total	550	47 (8.54)

### 3.2 *P. aeruginosa* antibiotic resistance pattern

Table 3 shows the *P. aeruginosa* antibiotic resistance pattern. *P. aeruginosa* isolates exhibited the maximum resistance rate toward penicillin (87.23%), ampicillin (85.10%), tetracycline (85.10%), gentamicin (65.95%) and trimethoprim (57.44%). The lowest resistance rate was found against chloramphenicol (6.38%), imipenem (17.02%), aztreonam (29.78%) and clindamycin (31.91%). *P. aeruginosa* strains isolated from carcass surface swab samples harboured a higher resistance rate toward all examined antibiotic agents (*p* < 0.05). Additionally, *P. aeruginosa* strains isolated from cattle samples harboured a higher resistance rate toward antibiotic agents (*p* < 0.05). Statistically significant differences were observed between types of samples and *P. aeruginosa* resistance rate (*p* < 0.05).

**TABLE 3:** *P. aeruginosa* antibiotic resistance pattern

	*N* (%) isolates resist to each antibiotic											
Samples (*N* positive)	C30*	T30	G10	S25	T5	CIP	P10	A10	CLN	Cef	AZ	Im10
Raw cattle meat (13)	1 (7.69)	11 (84.61)	9 (69.23)	5 (38.46)	6 (46.15)	4 (30.76)	11 (84.61)	9 (69.23)	3 (23.07)	5 (38.46)	3 (38.46)	2 (15.38)
Raw sheep meat (10)	–	7 (70)	4 (40)	4 (40)	3 (30)	3 (30)	7 (70)	8 (80)	2 (20)	2 (20)	2 (20)	1 (10)
Total (23)	1 (4.34)	18 (78.26)	13 (56.52)	9 (39.13)	10 (43.47)	7 (30.43)	18 (78.26)	17 (73.91)	5 (21.73)	7 (30.43)	5 (21.73)	3 (13.04)
Cattle carcass surface swab (14)	1 (7.14)	14 (100)	12 (85.71)	9 (64.28)	11 (78.57)	8 (57.14)	14 (100)	13 (92.85)	6 (42.85)	8 (57.14)	6 (42.85)	3 (21.42)
Sheep carcass surface swab (10)	1 (10)	8 (80)	6 (60)	6 (60)	6 (60)	3 (30)	9 (90)	10 (100)	4 (40)	5 (50)	3 (30)	2 (20)
Total (24)	2 (8.33)	22 (91.66)	18 (75)	15 (62.50)	17 (70.83)	11 (45.83)	23 (95.83)	23 (95.83)	10 (41.66)	13 (54.16)	9 (37.50)	5 (20.83)
Total (47)	3 (6.38)	40 (85.10)	31 (65.95)	24 (51.06)	27 (57.44)	18 (38.29)	41 (87.23)	40 (85.10)	15 (31.91)	20 (42.55)	14 (29.78)	8 (17.02)

* C30, chloramphenicol (30 µg/disk); T30, tetracycline (30 µg/disk); G10, gentamicin (10 µg/disk); S25, sulfamethoxazole (25 µg/disk); T5, trimethoprim (5 µg/disk); CIP, ciprofloxacin (5 µg/disk); P10, penicillin (10 µg/disk); A10, ampicillin (10 µg/disk); CLN, clindamycin (2 µg/disk); Cef, cefoxitin (10 µg/disk); AZ, aztreonam (30 µg/disk) and Im10, imipenem (10 µg/disk).

### 3.3 *P. aeruginosa* antibiotic resistance genes

Table 4 shows the distribution of antibiotic resistance genes among the examined *P. aeruginosa* isolates. *BlaCTX‐M*, *blaDHA* and *blaTEM* antibiotic resistance genes were found in 53.19, 42.55 and 27.65% of *P. aeruginosa* isolates. *BlaVEB*, *blaSHV* and *blaOXA* were only detected in 8.51, 10.63 and 17.02% of *P. aeruginosa* isolates. *P. aeruginosa* strains isolated from carcass surface swab samples harboured a higher distribution of antibiotic resistance genes than those isolates from meat samples (*p* < 0.05). Additionally, *P. aeruginosa* strains isolated from cattle samples harboured a higher distribution of antibiotic resistance genes (*p* < 0.05). Statistically significant differences were observed between types of samples and distribution of *P. aeruginosa* resistance genes (*p* < 0.05).

**TABLE 4:** Distribution of antibiotic resistance genes among the *P. aeruginosa* isolates

	N (%). isolates harboured each antibiotic resistance gene					
Samples (*N* positive)	*blaSHV*	*blaTEM*	*blaCTX‐M*	*blaOXA*	*blaVEB*	*blaDHA*
Raw cattle meat (13)	2 (15.38)	4 (30.76)	5 (38.46)	2 (15.38)	1 (7.69)	5 (38.46)
Raw sheep meat (10)	–	1 (10)	3 (30)	1 (10)	–	2 (20)
Total (23)	2 (8.69)	5 (21.73)	8 (34.78)	3 (13.04)	1 (4.34)	7 (30.43)
Cattle carcass surface swab (14)	2 (14.28)	5 (35.71)	12 (85.71)	3 (21.42)	2 (14.28)	8 (57.14)
Sheep carcass surface swab (10)	1 (10)	3 (30)	5 (50)	2 (20)	1 (10)	5 (50)
Total (24)	3 (12.50)	8 (33.33)	17 (70.83)	5 (20.83)	3 (12.50)	13 (54.16)
Total (47)	5 (10.63)	13 (27.65)	25 (53.19)	8 (17.02)	4 (8.51)	20 (42.55)

### 3.4 *P. aeruginosa* virulence factors

Table 5 shows the distribution of virulence factors among the *P. aeruginosa* isolates. *ExoS* (42.55%), *algD* (31.91%), *lasA* (31.91%), *plcH* (31.91%) and *exoU* (25.53%) were found to be the most commonly detected virulence factors. The distribution of *phzH* (2.12%), *exoY* (2.12%), *phzS* (4.25%) and *exoT* (6.38%) were lower than other detected virulence factors. *AlgU*, *plcN*, *phzM* and *lasB* virulence factors were detected in 14.89, 10.63, 8.51 and 8.51% of *P. aeruginosa* isolates, respectively. *P. aeruginosa* strains isolated from carcass surface swab samples harboured a higher distribution of virulence factors than those isolates from meat samples (*p* < 0.05). Additionally, *P. aeruginosa* strains isolated from cattle samples harboured a higher distribution of virulence factors (*p* < 0.05). Statistically significant differences were observed between types of samples and distribution of *P. aeruginosa* virulence factors (*p* < 0.05).

**TABLE 5:** Distribution of virulence factors among the *P. aeruginosa* isolates

Samples (*N* positive)	*N* (%) isolates harboured each virulence factor												
	*algD*	*algU*	*plcH*	*plcN*	*exoS*	*exoT*	*exoY*	*exoU*	*phzM*	*phzS*	*phzH*	*lasA*	*lasB*
Raw cattle meat (13)	4 (30.76)	2 (15.38)	4 (30.76)	1 (7.69)	4 (30.76)	1 (7.69)	–	2 (15.38)	1 (7.69)	–	–	3 (23.07)	1 (7.69)
Raw sheep meat (10)	2 (20)	–	2 (20)	1 (10)	3 (30)	–	–	2 (20)	–	–	–	2 (20)	–
Total (23)	6 (26.08)	2 (8.69)	6 (26.08)	2 (8.69)	7 (30.43)	1 (4.34)	–	4 (17.39)	1 (4.34)	–	–	5 (21.73)	1 (4.34)
Cattle carcass surface swab (14)	7 (50)	3 (21.42)	6 (42.85)	2 (14.28)	8 (57.14)	1 (7.14)	1 (7.14)	5 (35.71)	2 (14.28)	2 (14.28)	1 (7.14)	6 (42.85)	2 (14.28)
Sheep carcass surface swab (10)	4 (40)	2 (20)	3 (30)	1 (10)	5 (50)	1 (10)	–	3 (30)	1 (10)	–	–	4 (40)	1 (10)
Total (24)	11 (45.83)	5 (20.83)	9 (37.50)	3 (12.50)	13 (54.16)	2 (8.33)	1 (4.16)	8 (33.33)	3 (12.50)	2 (8.33)	1 (4.16)	10 (41.66)	3 (12.50)
Total (47)	17 (36.17)	7 (14.89)	15 (31.91)	5 (10.63)	20 (42.55)	3 (6.38)	1 (2.12)	12 (25.53)	4 (8.51)	2 (4.25)	1 (2.12)	15 (31.91)	4 (8.51)

### 3.5 Determination of multidrug‐resistant isolates

**FIGURE 3:**
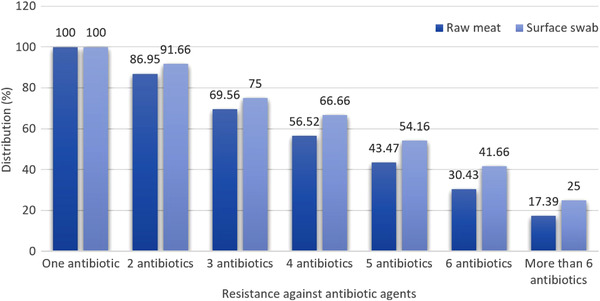
Distribution of MDR *P. aeruginosa* strains isolated from raw meat and surface swab samples

Figure 3 shows the distribution of multidrug‐resistant (MDR) *P. aeruginosa* strains isolated from raw meat and surface swab samples. According to obtained data, all *P. aeruginosa* isolates harboured complete resistance to at least one antibiotic agent (100%). The prevalence of resistance of *P. aeruginosa* isolates of raw meat samples against at least 3 and more than six antibiotic agents was 69.56 and 17.39%, respectively. Prevalence of resistance of *P. aeruginosa* isolates of carcass surface swab samples against at least three and more than six antibiotic agents were 76 and 25%, respectively.

## 4 DISCUSSION

Debating the origin of bacteria in food is very challenging. In the present study, samples of raw meat and swabs were taken from the surface carcasses of animal species that were contaminated with *P. aeruginosa* strains. In this regard, 6.57% of raw meat and 12% of carcass surface swab samples were contaminated with *P. aeruginosa* strains with a higher contamination rate for samples taken from cattle species. Because *P. aeruginosa* is so widespread in the environment and can sometimes be present on the surface of damaged human skin due to wounds or burns, the exact sources of the isolated strains may be environmental contamination or transmission through contaminated slaughterhouse personnel after meat and carcass manipulation. Additionally, *P. aeruginosa* strains are considered spoilage microorganisms in fresh meat. There is no probable reason for the higher prevalence of *P. aeruginosa* in samples taken from cattle. Scarce researches are available in this regard. In a survey by Sheir et al. (2020), 4.00% of meat product samples were contaminated with *P. aeruginosa* owing to the needless handling and manipulation of meat samples and poor hygienic quality of raw materials. A higher prevalence of *P. aeruginosa* in cattle meat samples was reported by Benie et al. (2017). They found that 53.04% of cattle meat was contaminated by *P. aeruginosa*. Similarly, Benie et al. (2016) reported that 97.90% of beef samples were positive for *P. aeruginosa* strains. Other surveys were conducted on chicken meat (Mahato et al., 2020), camel meat (Osman et al., 2019), cattle meat (Odoi et al., 2021), frozen meat (Ibrahim et al., 2016) and meat products (Sofy et al., 2017) samples showed that the *P. aeruginosa* prevalence had a range between 3.00 to 80.00%. High adaptation of *P. aeruginosa* strain in different temperatures (4–42°C) and water activities (72–97%) (Gu et al., 2016) may be another reason for its high prevalence among the examined samples.

Isolated *P. aeruginosa* strains harboured a high resistance rate toward diverse classes of antimicrobials. The highest resistance rates were obtained against penicillin, ampicillin, tetracycline, gentamicin and trimethoprim. Additionally, *P. aeruginosa* isolates harboured several antibiotic resistance genes, mainly *BlaCTX‐M*, *blaDHA* and *blaTEM*. Thus, phenotypically (disk diffusion) and genotypically (presence of the genes encoding resistance against antibiotics) *P. aeruginosa* isolates were resistant to antibiotic agents. Improper and unauthorised administration of antibiotics, overuse of antibiotics and disinfectants, and finally self‐medication with antibiotics can be possible reasons for the high prevalence of antibiotic resistance. The higher use of antibiotics in cattle than in sheep is a possible reason for the higher prevalence of antibiotic resistance in bovine isolates. Furthermore, contact of the carcass surface with the slaughterhouse environment and contaminated staff (which can carry *P. aeruginosa*) can cause the transfer of antibiotic‐resistant strains to the carcass surface of livestock. Therefore, it is logical that the prevalence of antibiotic resistance in strains isolated from the carcass surface is higher than those of meat samples. In a survey conducted in China (Meng et al., 2020), Australia (Khan et al., 2020), Germany (Yayan et al., 2015) and Saudi Arabia (Khan & Faiz, 2016), *P. aeruginosa* strains harboured a high resistance rate toward penicillin, ampicillin, tetracycline, gentamicin and trimethoprim antimicrobial agents. Benie et al. (2017) reported that *P. aeruginosa* strains isolated from raw meat samples harboured the highest resistance rate against kanamycin (100%), aztreonam (97.60%) and ciprofloxacin (35.40%). In a survey of Allydice‐Francis and Brown (2012), *P. aeruginosa* isolates harboured a high resistance rate toward ceftazidime (79.00%), ciprofloxacin (93.00%), gentamicin (97.00%) and imipenem (100%). Odumosu et al. (2016) reported that the prevalence of resistance against carbenicillin, amikacin and ceftazidime was 63.00, 1.90 and 83.30%, respectively. It seems that chloramphenicol, imipenem, aztreonam and clindamycin prescription may be effective against *P. aeruginosa* foodborne infections. ESBL‐producing P. aeruginosa isolates are one of the main antibiotic resistance‐related issues in the food industry (Mir et al., 2016). Our findings revealed that the genes that encode resistance toward cephalosprins (*blaShV*, *blaDHA* and *blaVEB*), penicillins (*blaCTX* and *blaDHA*), aztreonam (*blaTEM*) and carbapenems (*blaOXA*) were predominant among isolates. Similarly, Bahrami et al. (2018) described that *blaCTX‐M*, *blaSHV*, *blaTEM* and *blaOXA* were detected in 23.95, 23.08, 57.29 and 12.5% of *P. aeruginosa* strains, respectively. In a previous study (Elhariri et al., 2017), ESBL‐producing P. aeruginosa isolates of raw meat samples harboured *blaCTX‐M* (38.00%), *blaSHV* (33.30%) and *blaTEM* (28.50%) genes as predominant. Hosu et al. (2021) stated that *blaSHV*, *blaTEM* and *blaCTX‐M* antibiotic resistance genes among the *P. aeruginosa* isolates of non‐clinical samples were distributed 93.30, 40.00 and 20.00%, respectively. The high distribution of ESBL genes in the *P. aeruginosa* strains in this survey revealed the critical role of meat and carcass surface swab samples as a possible vehicle for the community‐wide dissemination of antimicrobial‐resistant *P. aeruginosa*, particularly ESBL‐producing type isolates.

Isolated strains also contained several putative virulence factors, mainly *exoS*, *algD*, *lasA*, *plcH* and *exoU*. These genes are mainly responsible for the adhesion and invasion of bacteria into the host cells. In keeping with this, consuming food containing virulent *P. aeruginosa* strains may cause severe foodborne infection. Similarly, *toxA*, *aprA*, *plcH* and *las* virulence factors were predominant in the *P. aeruginosa* strains isolated from raw meat (Osman et al., 2019). Benie et al. (2017) recognised that the distribution of *exoU*, *pilB*, *plcH*, *algD*, *exoS* and *lasB* virulence factors in the *P. aeruginosa* strains isolated from bovine meat samples were 4.10, 42.60, 72.10, 74.50, 96.70 and 96.70%, respectively. It seems that *P. aeruginosa* isolates of meat and animal carcass samples in this survey can cause severe gastrointestinal disorders owing to the high distribution of virulence factors. However, some additional work should perform to approve it.

In conclusion, *P. aeruginosa* strains were detected in 8.54% of raw meat and carcass surface swab samples. The considerable prevalence of *P. aeruginosa* strains was accompanied by the high rate of bacterial resistance toward commonly used antibiotic agents, particularly penicillin, ampicillin, tetracycline, gentamicin and trimethoprim. The findings may show the high antibiotic resistance of *P. aeruginosa* and the potential role of raw meat and carcass surface swab samples in its transmission to the human population. Some strains harboured different antibiotic resistance genes, particularly *blaCTX‐M*, *blaDHA* and *blaTEM*, and virulence factors, especially *ExoS*, *algD*, *lasA*, *plcH* and *exoU*. These findings may show the role of raw meat and carcass surface swab samples as a source of virulence factors and antibiotic resistance genes. It seems that the consumption of contaminated meat with virulent and resistant *P. aeruginosa* may cause severe foodborne diseases that resist antibiotic therapy. However, the role of contaminated meat as a hazard of foodborne infection has not been determined yet. Thus, several studies should perform to assess the role of meat and animal carcasses in the transmission of virulent and resistant *P. aeruginosa* foodborne diseases.

Samples collected from carcass surface swab samples harboured a higher prevalence rate, antibiotic resistance rate and distribution of virulence factors. This may show the higher contamination rate of animal carcass surfaces with resistant and virulent bacteria. As samples were collected at the end of the slaughter procedure (after washing), it seems that the washing of animal carcasses was not done properly. As isolates of carcass surface swab samples harboured higher antibiotic resistance, it may conclude that some isolates may transfer after carcass manipulation by meat inspectors and staff of the slaughterhouses.

The present study was limited to the low diversity of collected samples, especially the lack of samples collected from ovine, camel and buffalo species. The absence of molecular typing of *P. aeruginosa* isolates was another important limitation of this survey. An adequate number of collected samples, phenotypically and genotypically determination of antibiotic resistance patterns of *P. aeruginosa* isolates, and finally an examination of the distribution of MDR isolates were the most important strong points of this survey.

## AUTHOR CONTRIBUTION

*Conceptualisation*: Mohammad Ahmadi, Shahrokh Poursina; *Data curation*: Fatemeh Fazeli, Peiman Ariaii; *Formal analysis*: Mohammad Ahmadi, Shahrokh Poursina; *Funding acquisition*: Mohammad Ahmadi, Shahrokh Poursina; *Investigation*: Fatemeh Fazeli, Peiman Ariaii; *Methodology*: Shahrokh Poursina; *Resources*: Mohammad Ahmadi, Shahrokh Poursina; *Software*: Fatemeh Fazeli, Peiman Ariaii; *Supervision*: Mohammad Ahmadi; *Validation*: Mohammad Ahmadi, Shahrokh Poursina, Fatemeh Fazeli, Peiman Ariaii; *Writing – original draft and writing – review and editing*: Mohammad Ahmadi.

## CONFLICT OF INTEREST

The authors declared that they have no conflict of interest.

## ETHICS STATEMENT

This study was reviewed and approved by the Research Ethics Committee of the College of Veterinary Medicine, Islamic Azad University. Owners, managers and workers of the different sites were informed of the procedures and significance of the study. Each data and analysis result was kept confidential and communicated to concerned bodies. Any participants who were not volunteers were not forced to be included.

### PEER REVIEW

I would not like my name to appear with my report on Publons https://publons.com/publon/10.1002/vms3.1007.

## Data Availability

Data will available upon request from the authors.
